# Unlocking the Complete Chloroplast Genome of a Native Tree Species from the Amazon Basin, Capirona (*Calycophyllum Spruceanum*, Rubiaceae), and Its Comparative Analysis with Other Ixoroideae Species

**DOI:** 10.3390/genes13010113

**Published:** 2022-01-07

**Authors:** Carla L. Saldaña, Pedro Rodriguez-Grados, Julio C. Chávez-Galarza, Shefferson Feijoo, Juan Carlos Guerrero-Abad, Héctor V. Vásquez, Jorge L. Maicelo, Jorge H. Jhoncon, Carlos I. Arbizu

**Affiliations:** 1 Dirección de Desarrollo Tecnológico Agrario, Instituto Nacional de Innovación Agraria (INIA), Av. La Molina 1981, Lima 15024, Peru; carla18317@gmail.com (C.L.S.); pmrg1711@gmail.com (P.R.-G.); jcchavezgalarza@gmail.com (J.C.C.-G.); hvasquez@inia.gob.pe (H.V.V.); jmaicelo@inia.gob.pe (J.L.M.); 2 Facultad de Ciencias, Universidad Nacional José Faustino Sánchez Carrión, Av. Mercedes Indacochea Nro. 609, Huacho 15136, Peru; 3 Estación Experimental Agraria San Bernardo, Dirección de Desarrollo Tecnológico Agrario, Instituto Nacional de Innovación Agraria (INIA), Carretera Cusco, Puerto Maldonado, Tambopata, Madre de Dios 17000, Peru; sfeijoo@inia.gob.pe; 4 Dirección de Recursos Genéticos y Biotecnología, Instituto Nacional de Innovación Agraria (INIA), Av. La Molina 1981, Lima 15024, Peru; jguerreroa@inia.gob.pe; 5 Centro de Investigación de Plantas Andinas y Nativas, Facultad de Ciencias, Universidad Nacional de Educación Enrique Guzmán y Valle, Av. Enrique Guzmán y Valle s/n, Lima 15472, Peru; jjhoncon@une.edu.pe; 6 Unidad de Investigación, Perú Maca SAC, Panamericana Sur KM. 37.2 Mz. D1. Lote 03A, Lima 15823, Peru

**Keywords:** chloroplast, genetic resources, genomics, capirona, phylogenomics

## Abstract

Capirona (*Calycophyllum spruceanum* Benth.) belongs to subfamily Ixoroideae, one of the major lineages in the Rubiaceae family, and is an important timber tree. It originated in the Amazon Basin and has widespread distribution in Bolivia, Peru, Colombia, and Brazil. In this study, we obtained the first complete chloroplast (cp) genome of capirona from the department of Madre de Dios located in the Peruvian Amazon. High-quality genomic DNA was used to construct libraries. Pair-end clean reads were obtained by PE 150 library and the Illumina HiSeq 2500 platform. The complete cp genome of *C. spruceanum* has a 154,480 bp in length with typical quadripartite structure, containing a large single copy (LSC) region (84,813 bp) and a small single-copy (SSC) region (18,101 bp), separated by two inverted repeat (IR) regions (25,783 bp). The annotation of *C. spruceanum* cp genome predicted 87 protein-coding genes (CDS), 8 ribosomal RNA (rRNA) genes, 37 transfer RNA (tRNA) genes, and one pseudogene. A total of 41 simple sequence repeats (SSR) of this cp genome were divided into mononucleotides (29), dinucleotides (5), trinucleotides (3), and tetranucleotides (4). Most of these repeats were distributed in the noncoding regions. Whole chloroplast genome comparison with the other six Ixoroideae species revealed that the small single copy and large single copy regions showed more divergence than inverted regions. Finally, phylogenetic analyses resolved that *C. spruceanum* is a sister species to *Emmenopterys henryi* and confirms its position within the subfamily Ixoroideae. This study reports for the first time the genome organization, gene content, and structural features of the chloroplast genome of *C. spruceanum*, providing valuable information for genetic and evolutionary studies in the genus *Calycophyllum* and beyond.

## 1. Introduction

The family Rubiaceae is one of the largest and most diverse families of angiosperms, and includes the economically important genus *Coffea* and the horticulturally important *Gardenia* and *Ixora*, all part of Ixoroideae subfamily [1,2]. This subfamily comprises about 4000 species of pantropical and subtropical distributions and is one of the three major lineages in the Rubiaceae family, and it includes *Coffea canephora*, *Fosbergia shweliensis*, *Scyphiphora hydrophyllacea*, *Emmenopterys henryi*, and *Calycophyllum spruceanum* “capirona” [1]. Capirona is an important timber tree [3], with its origin in the Amazon Basin and widespread distribution in Bolivia, Peru, Colombia, and Brazil [4]. It is a rainforest hardwood tree and is also exported around the world for high density wood, durable lumber and building materials, and as a medicinal plant. Moreover, it is used for the construction of economically valuable products [5], including construction poles, firewood, and charcoal [6]. It has excellent qualities for field planting or in agroforestry system combinations. In addition, capirona has good natural regeneration and is an ideal species for the management of secondary successions [3]. Secondary succession occurs when woody vegetation grows back after complete clearing of the forest for pasture, agriculture, or other human activities such as logging for pulp or wood [7]. However, to date, *C. spruceanum* is considered a neglected forest species as genetic and genomic resources for this species are still limited. Very few molecular studies have been conducted for this forest species. Russell et al. [3], Tauchen et al. [5], and Saldaña et al. [8] determined the genetic variation of capirona using molecular markers, such as amplified fragment length polymorphisms (AFLP), internal transcribed spacer (ITS), and random-amplified polymorphic DNA (RAPD), respectively, in different populations of capirona from the Peruvian Amazon. Their results demonstrated a greater variation within provenances than among them. In contrast, Dávila-Lara et al. [9] used AFLP and reported low genetic diversity parameters across 13 populations of capirona in Nicaragua (Central America). To date, SSR markers were not developed in capirona. SSR markers are codominant, highly polymorphic, reproducible, reliable, and distributed throughout the genome, and they are widely used in assessing the genetic diversity and population structure [10] of economically important forest species, such as red oak [11], Chinese white poplar [12], and American cedar [13]. Genetic diversity studies are indispensable for conducting conservation programs and sustainable management. Studies based on molecular markers provide important information on the genetic makeup of the population because they are independent of environmental factors [14]. Capirona is attracting the attention of many investigators in the Peruvian Amazon basin, in the context of increased deforestation through unsustainable slash and burn agriculture, and also for conservation strategies [3].

Chloroplasts, as metabolic organelles responsible for photosynthesis and the synthesis of amino acids, nucleotides, fatty acids, phytohormones, vitamins, and other metabolites, play an important role in the physiology and development of land plants and algae [15,16]. They have their own genetic replication mechanisms, and they transcribe their own genome relatively independently [17]. In most terrestrial plants, chloroplast genomes possess highly conserved and organized structures, occur as circular DNA molecules with a size of 120–170 kb [18], and have a highly conserved quadripartite structure and normally encodes approximately 110–130 genes involved in photosynthesis, transcription, and translation processes. In addition, chloroplast genomes contain two inverted repeat sequences (IR), as well as a large single copy region (LSC) and a small single copy region (SSC) [19,20]. Although the chloroplast genomes of angiosperms are highly conserved, mutational events occur, such as structural rearrangement, insertions, and deletions, inversions, translocations, and variations in the number of copies. This polymorphism in the chloroplast genome provides valuable information about population genetics and structure, phylogeny, species barcode analysis, and endangered species conservation and breeding improvement [21]. In addition, the chloroplast genome will provide us information about the codon usage bias. It allows us to evaluate the preference for certain synonymous codons during translation of genes in all genomes examined [22]. Thus, the coding sequences of a genome are the blueprints of gene products that provide valuable information on gene function and evolution of the organism [23].

To date, there is no report on the application of whole genomic sequencing techniques to study *Calycophyllum* spp. genomes. We here present the first complete chloroplast genome sequence of *C. spruceanum* based on the Illumina sequencing technology. A comparative analysis of *C. spruceanum* with six closely related species that belong to the Ixoroideae subfamily is reported. Our study provided useful information on genome organization, gene content, and structure variation in the *C. spruceanum* chloroplast genome, and also provided important clues to its phylogenetic relationships, which will contribute to genetic and evolutionary studies in *C. spruceanum* and beyond.

## 2. Materials and Methods

### 2.1. Plant Materials and Genomic DNA Extraction

A single capirona tree was selected to be sequenced from San Bernardo Research Station of INIA, located in Madre de Dios department (2°41′8.66″ N/69°22′49.8″ E/227.2 m.a.s.l) in the Peruvian Amazon. A branch with flowers was collected and deposited at the Scientific Collection of the Herbarium of Universidad Nacional Mayor de San Marcos (UNMSM), under the voucher number 324323. Total genomic DNA was extracted from fresh leaves by the CTAB method [24], with minor modifications according to the protocol of Cruz et al. [25]. The quality was evaluated on a 1% agarose gel and the quantification was performed by fluorescence using the Qubit™ 4 Fluorometer (Invitrogen, Waltham, MA, USA), according to the Qubit 4 Quick Reference Guide.

### 2.2. DNA Sequence and Genome Assembly

High-quality genomic DNA was used to construct libraries. Pair-end (PE) clean reads were obtained by the Illumina HiSeq 2500 platform and PE 150 library using the NexteraXT DNA Library Preparation Kit (Illumina, San Diego, CA, USA). Adapters and low-quality reads were removed using Trim Galore [26] with default settings. We used clean data, and similar to Arbizu et al. [27], *Coffea arabica* (NC_008535) was used as reference to assemble the chloroplast genome employing the GetOrganelle v1.7.2 pipeline [28] with the following arguments: *−F* embplant_pt *−R* 15 *–reduce-reads-for-coverage* inf. SPAdes v3.11.1 [29], bowtie2 v2.4.2 [30], and BLAST+ v2.11 [31] were also employed with default settings within this pipeline. The accuracy of the assembled chloroplast (cp) genome and its read depth were confirmed by mapping the short reads to the capirona assembled cp genome using Burrows-Wheeler Aligner (BWA) software [32], and the plot was created using *ggplot*2 v3.3.5 package [33] in R software v4.0.2 [34].

### 2.3. Annotation and Analysis of C. spruceanum Chloroplast DNA Sequence

The annotations of the protein-coding genes (PCGs), transfer RNAs (tRNAs), and rRNA genes from *C. spruceanum* chloroplast genome were performed using webserver Geseq [35] with default settings by comparing to all available plastid genomes in NCBI of Ixoroideae associated with this server and curated manually. The codon usage analysis was carried out with MEGA X software [36]. The architecture of *C. spruceanum* chloroplast genome was visualized using OGDRAW 1.3.1 [37].

### 2.4. Comparative Analysis of Ixoroideae Chloroplast Genomes

The Shuffle-LAGAN mode of the mVISTA online program (http://genome.lbl.gov/vista/mvista/ accessed on 13 October 2021) [38] was used to compare the sequence similarity of the complete chloroplast genome of *Calycophyllum spruceanum* with six species of Ixoroideae sub family (Table 1). The annotated *C. spruceanum* chloroplast genome generated in this work was used as reference. An identity matrix was generated; previously independent alignments of each of the regions were done using MAFFT v7.475 software [39] considering the “auto” argument, that is, the software automatically selects an appropriate strategy, according to data size. Further manual alignment corrections were performed using MacClade v4.08a [40]. The identity plot was generated using the ggplot2 package in the R software. Extension packages were also used, including ggtext (https://github.com/wilkelab/ggtext/issues accessed on 22 December 2021) and ggpubr [41]. This identity matrix clearly shows which genomes have greater identities.

SSRs within the *C. spruceanum* chloroplast genome were searched using the MISA software [42]. The criteria of SSR research were set as follows: the minimum numbers of repeats for mononucleotides, dinucleotides, trinucleotides, tetranucleotides, pentanucleotides, and hexanucleotides were 10, 5, 4, 3, 3, and 3, respectively [43]. A plot with the structure and location of the SSRs in the seven cp genomes analyzed in this study was generated using the *genoPlotR* [44] and *gggenomes* (https://github.com/thackl/gggenomes accessed on 22 December 2021) packages in the R software. The codon usage, frequency, and relative synonymous codon usage (RCSU) of the *C. spruceanum* cp genome were analyzed using MEGA X software [36]. The parameters used were set to default.

### 2.5. Phylogenetic Analyses

To gain an insight into the phylogenetic location of *C. spruceanum*, a maximum-likelihood (ML) tree was constructed with 1000 nonparametric bootstrap replicates using RAxML v8.2.11 software [45] under the GTR + γ nucleotide substitution model of evolution. The complete chloroplast genome of *C. spruceanum* was compared and aligned by the MAFFT software [39] with the other 19 chloroplast genomes obtained from Genbank. Seven species from Rubioideae, five species from Cinchonoideae, and six species from Ixoroideae were included in the analysis. We used all Rubiaceae species chloroplast genomes that were available at Genbank (https://www.ncbi.nlm.nih.gov/genome/browse#!/organelles/rubiaceae accessed on 9 September 2021). *Lonicera hispida* (Caprifoliaceae) was included as an outgroup. We conducted a Bayesian analysis considering two independent four-chain 50 million generation runs per input file and sampling every 1000 generations. Tracer v1.6 (http://tree.bio.ed.ac.uk/software/tracer/ accessed on 23 December 2021) was used to analyze the convergence to the stationary distribution and the effective sample size (ESS) of each parameter of each input file. We discarded the first 25% of generations as burn-in. The resulting tree was viewed in FigTree version 1.4.4 (http://tree.bio.ed.ac.uk/software/figtree/ accessed on 24 December 2021).

## 3. Results

### 3.1. C. spruceanum Chloroplast Genome Assembly and Its Features

The overall length of the *C. spruceanum* chloroplast genome is 154,480 bp, exhibiting the circular quadripartite structure characteristic of major angiosperm plants. After annotation and modification, the entire chloroplast (cp) genome sequence was submitted to the GenBank database with accession number: OK326865 (https://www.ncbi.nlm.nih.gov/nuccore/OK326865.1/ accessed on 5 January 2022). The associated Bioproject, Biosample, and SRA numbers are PRJNA760977, SAMN21240132, and SRR15725575, respectively. The chloroplast genome assembled exhibited an average coverage depth of 449X (Figure S1).

**Figure 1:**
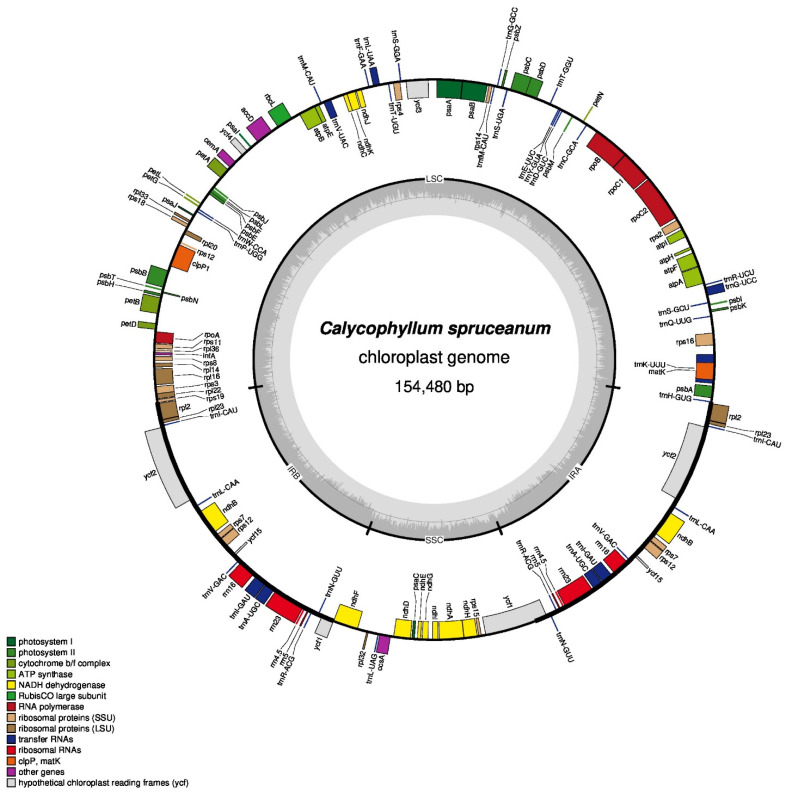
Gene map of *C. spruceanum*. Genes lying outside the outer circle are transcribed in a counter-clockwise direction, and genes inside this circle are transcribed in a clockwise direction. The colored bars indicate known protein-coding genes, transfer RNA genes, and ribosomal RNA genes. LSC, large single-copy; SSC, small single-copy; IR, inverted repeat.

The chloroplast genome of capirona consists of a pair of the inverted repeat (IR) regions (25,783 bp) separated by a large single-copy (LSC) region of 84,813 bp and a small single-copy (SSC) region of 18,101 bp. A circular representation of the complete chloroplast genome is shown in Figure 1. The GC content of the IR region (43.14%) was much higher than that of the LSC (31.89%) and SSC regions (35.48%) in the *C. spruceanum* cp genome (Table 1). The annotation of cp genome predicted a total of 133 genes, of which 114 are unique, consisting of 80 protein-coding genes, 30 transfer RNA (tRNA) genes, four ribosomal RNA (rRNA) genes, and one pseudogene (Table S1). Of these, seven protein-coding genes, four rRNAs, and seven tRNAs are duplicated in the IR regions. A total of 10 protein-coding genes and eight tRNAs genes contained a single intron, whereas three genes exhibited two introns each. The rps12 gene was predicted to be trans-spliced with its 5′ end located at the LSC region and the 3′ end with a copy located in each of the two IR regions.

As expected, the duplicated IR of the *C. spruceanum* chloroplast genome resulted in complete duplication of 18 genes: five protein-coding genes such as *rpl2*, *rpl23*, *rps7*, *rps12*, and *ndhB*; seven tRNAs as *trn*I-CAU, *trn*L-CAA, *trn*V-GAC, *trn*I-GAU, *trn*A-UGC, *trn*R-AGC, and *trn*N-GUU; four rRNAs genes as *rrn*23, *rrn*16, *rrn*5, *rrn*4.5 (see Figure 1), and 5′ end of *ycf1*. The SSC region contained 12 protein-coding and one tRNA gene, whereas LSC region contained 69 protein-coding and 22 tRNAs.

Codon usage analysis identified a total of 26,572 codons in the *C. spruceanum* chloroplast genome. Among all codons, leucine (Leu) was the most abundant amino acid with a frequency of 10.62%, followed by isoleucine (Ile) with a frequency of 8.40%, whereas cysteine (Cys) was less abundant with a frequency of 1.14%. Moreover, only one codon was identified for methionine (Met) and tryptophan (Trp) amino acids. Thirty codons were observed to be used more frequently than the expected usage at equilibrium (RSCU > 1), and 31 codons showed the codon usage bias: (RSCU < 1) and the third positions of the biased codons were A/U (Table S2). Biased codons with the highest values of RSCU were Leu (UUA), Ser (UCU), Gly (GGA), Tyr (UAU), and Asp (GAU).

### 3.2. Comparative Analysis of Genome Structure

**Figure 2:**
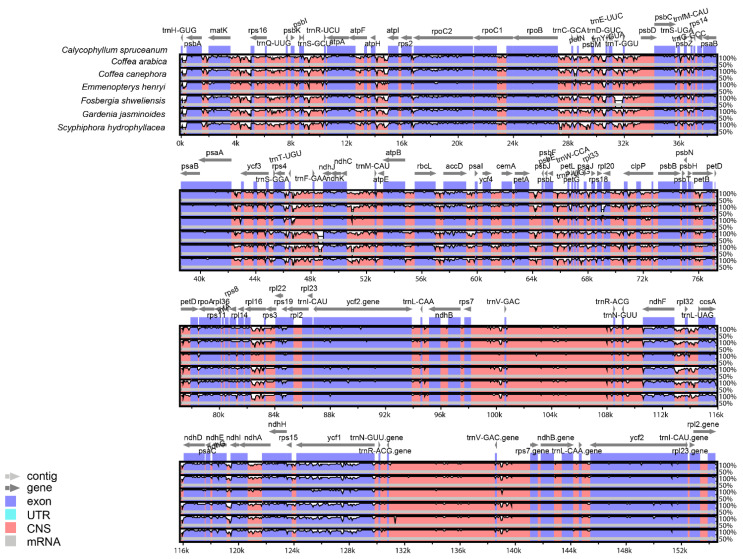
mVISTA identity plot comparing the seven Ixoroideae plastid genomes considering *C. spruceanum* as a reference. The top line shows genes in order (transcriptional direction indicated by arrows). The *y*-axis represents the percent identity within 50–100%. The *x*-axis represents the coordinate in the chloroplast genome. Genome regions are color-coded as protein-coding (exon), tRNAs, or rRNAs, and conserved noncoding sequences (intergenic region). The white block represents regions with sequence variation between two species.

In order to determine the structural characteristics of the *C. spruceanum* chloroplast genome (154,480 bp total length), we compared it with the other six Ixoroideae species: *Coffea canephora*, *C. arabica*, *F. shweliensis*, *S. hydrophyllacea*, *E. henryi*, and *G. jasminoides*, whose chloroplast genome differs in 271 bp, 709 bp, 237 pb, 652 bp, 899 bp, and 441 bp, respectively. Table 1 shows the genome size of each species. Our results showed that gene coding regions were more conserved than the noncoding regions, and the SSC and LSC regions showed more divergence than the IRa and IRb regions (Figure 2 and Figure S2). Additionally, it was also observed that the intergenic spacers regions between several pairs of genes varied greatly, for example, between *psbA-trnH-GUG*, *rps16-matK*, *atpI-atpH*, *ndhJ-rps4*, *rbcL-psaI*, *psaI-petA*, *ycf11-rps15* and *rpl32-ndhF*. In the coding regions, slight variations in sequence were observed in *matK*, *rpoC2*, *rps19*, and *ycf1* (Figure 2). The identity matrix revealed that the values in the IR region varied from 0.91 to 0.99. The LSC region presented values that fluctuated from 0.90 to 0.97, and the SSC region presented the highest divergence values, ranging from 0.82 to 0.97 (Figure S2). Gene order between *C. spruceanum* and other six Ixoroideae species showed similar patterns; however, greater divergences were found between *C. spruceanum* and *C. canephora*.

### 3.3. SSR Loci Identified in Ixoroideae cp Genomes

**Figure 3:**
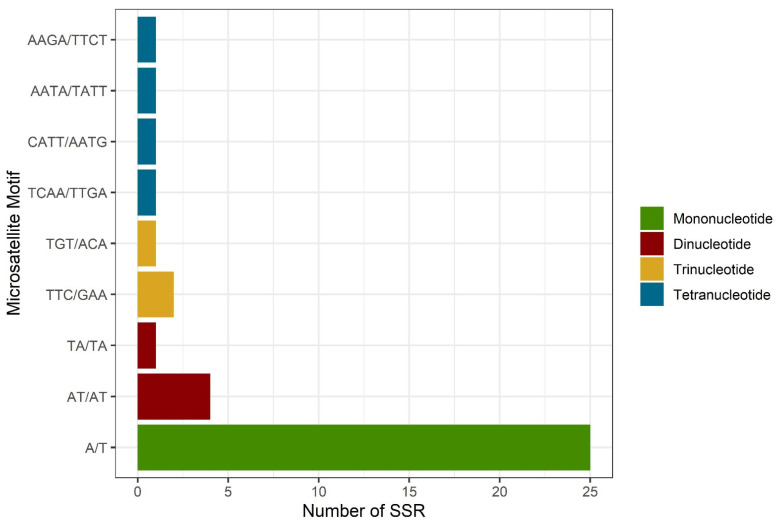
Analysis of simple sequence repeats (SSRs) distribution in *C. spruceanum*. The *x*-axis shows the number of SSRs. The *y*-axis shows SSR motif. The colored bars indicate the different repeats within SSRs.

The analysis of SSRs distribution within the *C. spruceanum* chloroplast genome revealed a total of 41 SSRs. The most abundant were the mononucleotide repeats (29) followed by dinucleotides (5). Additionally, SSRs with trinucleotides repeats (3) and tetranucleotides repeats (4) motifs in these genomes were identified in lower quantities (Figure 3). The number of SSRs identified for *C. arabica*, *C. canephora*, *F. shweliensis*, *S. hydrophyllacea*, *E. henryi*, and *G. jasminoides* was variable (43, 38, 42, 52, 46, and 30, respectively) (Table S3). All of these species presented the highest number of SSRs for A/T mononucleotides and for AT/TA dinucleotides. Only *F. shweliensis* and *S. hydrophyllaceae* presented SSRs with pentanucleotide repeats, and even *S. hydrophyllaceae* has SSRs with hexanucleotide repeats. Moreover, we detected that the SSRs were not only found in the non-coding regions (*psbA-trnH-GUG*, *rps16- matK*, *atpI-atpH*, *ndhJ-rps4*, *rbcL- psaI)*, but also in coding regions, such as *rpoC2* and ycF2, *ndhF*, *ndhG*, and *matK*. Also, we detected SSRs located in tRNA sequences in lower quantities (Figure S3).

### 3.4. Phylogenetic Inference of C. spruceanum

**Figure 4:**
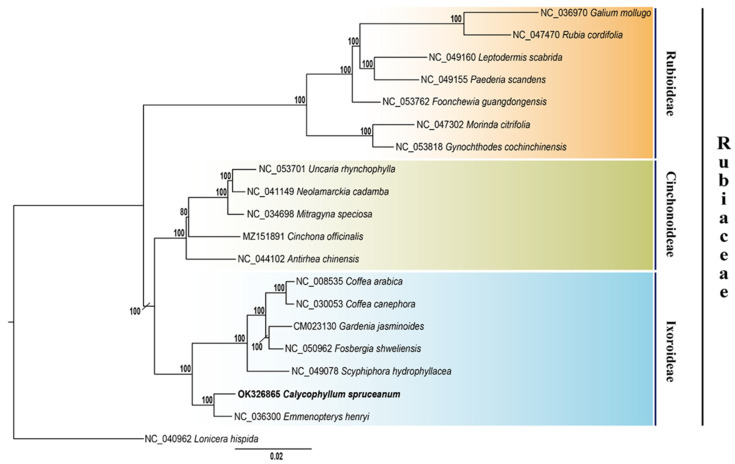
The maximum likelihood (ML) phylogenetic tree of the Rubiaceae family based on chloroplast genome sequences. Values along branches correspond to bootstrap percentages. The position of capirona (*C. spruceanum*) is indicated in black text. *Lonicera hispida* was set as the outgroup.

In this study, 19 species belonging to Rubiaceae and one outgroup (*Lonicera hispida*, Caprifoliaceae) were employed to infer their phylogenetic relationships using complete chloroplast genome sequences. Alignments were deposited into Dryad (https://datadryad.org/stash/share/1NWVfzxB6z6WZPEMAM0yAfzN5bl9L_8Uup2Z1WlbMu4 accessed on 31 December 2021). Maximum likelihood (ML) phylogenetic tree topology revealed well-supported monophylies for subfamilies Rubioideae, Cinchonoideae, and Ixoroideae. ML bootstrap support (BS) were very high: 16 nodes had 100% bootstrap values, and only one presented 80%. As expected, *C. spruceanum* was placed within subfamily Ixoroideae, and with 100% BS revealed to be a sister species of *Emmenopterys henryi* (Figure 4). Our Bayesian tree was very similar to the ML tree topology; all nodes presented a posterior probability of 1 (Figure S4). These phylogenetic trees were consistent with traditional taxonomy of the Rubiaceae family.

## 4. Discussion

Until very recently, only a few complete chloroplast genome sequences for the Ixoroideae subfamily were deposited into GenBank, with the very first being that of *Coffea canephora* in 2016. Nevertheless, with the development of next generation sequencing (NGS), the chloroplast (cp) genome of most species of the Ixoroideae subfamily has been obtained [2,43,46,47]. However, to date, cp genome of members of the genus *Calycophyllum* remained unknown. Thus, in the present study we sequenced for the first time the *C. spruceanum* chloroplast genome (accession number: OK326865.1) and compared it with other members of the subfamily Ixoroideae that are closely related. The *C. spruceanum* cp genome agrees with the characteristics of most angiosperm species in structure and gene content. The complete cp genome of *C. spruceanum* was 154,480 pb, similar to other Ixoroideae genomes [46,47], with a quadripartite structure (LSC, SSC, and two IR regions), which is a common characteristic in higher plants [11]. The annotation of *C. spruceanum* cp genome predicted 87 protein-coding genes (CDS), and similar patterns of protein-coding genes are also present in other Rubiaceae plants [43]. Similar to other studies [27,48], there were three genes (*rps12*, *clpP1*, and *ycf3*) that included two intron regions in the cp genome of capirona. It has been demonstrated that gene *clpP1* (caseinolytic protease P1) is essential for plant development [49] and function of plastids with active gene expression [50,51]. Moreover, Boudreau et al. [52] demonstrated that gene *ycf3* is required for the accumulation of the photosystem I (PSI) complex, interacting with the PSI subunits at a post-translational level [53]. Studies on these genes are needed, as they will contribute to the investigation of chloroplast in *C. spruceanum*.

Guanine-cytosine (GC) content has been a very useful tool to characterize in general terms the behavior of genomes [54,55].The GC content in the IR region was much higher than in the LSC and SSC regions in the *C. spruceanum* cp genome, probably due to the presence of eight ribosomal RNA (rRNA) genes in this region, which is consistent with previous analyses in other Ixoroideae [43,46] and in other angiosperms cp genomes [21,56,57]. The IR (A/B) region has always been considered consistent and stable in the cp genome, and it is also common in the evolution of plants with contraction or expansion events in the border region [43]. Also, these results suggest that the cp genome in this subfamily had rather conserved genome organization [43,46]. We identified that in the seven sequences of the cp genome are some highly divergent regions, including *psbA-trnH-GUG*, *rps16-matK*, *atpI-atpH*, *ndhJ-rps4*, *rbcL-psaI*, *psaI-petA*, *ycf1-rps15*, and *rpl32-ndhF*. These variable regions could be used for the development of molecular markers for DNA barcoding and phylogenetic studies in species of the Ixoroideae subfamily. Interestingly, *C. canephora* presents higher divergence values when compared with the other six species (Figure S2). The high divergence between *C. canephora* and other Ixoroideae chloroplast genomes could be due to biological events such as inversions, deletions, insertions, or genomic rearrangements [57,58]. Further research is needed to determine the exact cause of this divergence. In addition, the *ycf1* gene presented the greatest differentiation, suggesting that it is useful for providing phylogenetic resolution at the species level, as demonstrated for genus *Pinus* and *Daucus* [59,60].

We identified simple sequence repeats (SSRs), also known as microsatellites, in *C. spruceanum*. They are powerful molecular markers and are widely used to assess genetic diversity, population structure, evolutionary studies chloroplast genome rearrangement, and recombination processes [61,62,63] due their abundant polymorphism, high stability, codominant inheritance, and ease of use [64]. In addition, SSRs have been widely applied as molecular markers because of their unique uniparental inheritance [10,65]. In total, 41 perfect SSRs were detected in *C. spruceanum* cp genome distributed in the LSC, SSC, and IR regions with strong A/T bias. Similarly, previous studies also revealed that the non-coding region contained more SSRs than the coding regions [21,43]. Our results are also comparable to those of several previous studies showing that SSRs in cp genomes are highly rich in polythymine (poly T) or polyadenine (polyA) [66,67,68]. In contrast, repeats containing tandem cytosine (C) and guanine (G) were limited. Our results are in agreement with other studies that report microsatellites markers for other Ixoroideae species such as *C. arabica*, *C. canephora*, and *E. henryi* [43]. However, our results differ from those obtained by Wang et al. [46] for *G. jasminoides*. They identified only two SSRs, mono and di-nucleotide categories. In addition, they obtained 25 mononucleotide repeats and two of dinucleotides. We report 41 SSRs, the mononucleotide repeats (29) being the most abundant, followed by dinucleotides (5). Additionally, SSRs with trinucleotides repeats (3) and tetranucleotides repeats (4) motifs in these genomes were identified in lower quantities. With the identification of the SSR in the cp genome of *C. spruceanum*, we will be able to evaluate the polymorphism at the intraspecific level, as well as to evaluate the genetic diversity between and within the populations of *C. spruceanum*. These markers could also be used to aid in the selection and characterization of genotypes plus they are suitable for the development of a modern genetic improvement and conservation program.

Codon usage bias is a known phenomenon that occurs in a wide variety of organisms. Reporting codon use bias for the first time in capirona gives us important information about gene expression level, mutation frequency, GC composition, and abundance of tRNA [69,70]. Further understanding of codon preference facilitates the determination of optimal codons and the design of vectors in chloroplast genetic engineering [19]. Apparently, the major cause for selection on codon bias is that some preferred codons are translated more efficiently [71]. As reported for other chloroplast genomes of plants [72], our study revealed the preference in the use of synonymous codons, and the RSCU values of 30 codons resulted in >1 with biased codons in the third positions for A/T, which may be originated by a composition bias for a high A/T ratio [68]. These results are in accordance with other studies, where the codon usage preference for A/T is found in most other land plant chloroplast genomes [73]. Gene expression and the molecular evolution system of *C. spruceanum* may be elucidated by conducting research on its codon usage.

The rapid progress in the field of chloroplast genetics and genomics has been facilitated by the advent of high-throughput sequencing technologies. Chloroplast genomes have many features that make them useful for phylogenetic studies, resolving evolutionary relationships within phylogenetic clades, especially at low taxonomic levels [59,74,75]. Our entire plastid analysis of Rubiaceae provided a highly supported topology of the family, as reported by Bremer and Eriksson [76], using five chloroplast regions by Bayesian analysis. Similar to their work, it was possible to obtain very high bootstrap support (BS) for the three subfamilies (Cinchonoideae, Rubioideae, Ixoroideae) clades. Similar to Bremer and Eriksson [76], the availability of the complete *C. spruceanum* chloroplast genome allowed us to confirm the phylogenetic position of this forest tree species among Rubiaceae, suggesting that the chloroplast genome sequences can effectively resolve relationships of species, as demonstrated by Spooner et al. [59] and Bedoya et al. [77] for *Daucus* and Podostemaceae, respectively. With 100% BS, *C. spruceanum* was placed as sister species to *Emmenopterys henryi* within the Ixoroideae subfamily, confirming its classification within the Condomineae tribe, as suggested by previous studies based on a reduced number of genes and morphological data [1,78]. However, employing additional members of the subfamily Ixoroideae as well as nuclear genome sequences would provide more evidence to accurately infer the evolution history of *Calycophyllum*.

## 5. Conclusions

Here, we first reported the complete chloroplast genome sequence of a forest tree species, *C. spruceanum*, and a comparative analysis of six Ixoroideae cp genomes to reveal their genome features. We identified 41 SSRs that can be used for breeding, population genetics, and evolutionary studies. The genome structure, gene order, and content were found to be much conserved for all species; however, *C. canephora* presented higher divergence values when compared with the other six species. Both the LSC and SSC regions were more divergent than the IR region in the chloroplast genome of *C. spruceanum* compared to the other species, with the two most variable regions (*PsbA-rps16*) found in the LSC region. Furthermore, the phylogenomic analysis based on whole cp genomes generated ML and Bayesian trees with the same topologies as previously reported by other researchers, consolidating the taxonomical position of *C. spruceanum* species within the Ixoroideae subfamily and Condomineae tribe. These results provided important information on the genome organization, gene content, and structural variation of capirona and other Ixoroideae cp genomes. In addition, this new molecular resource will definitely help in the conservation of this native tree species from the Amazon basin.

## Figures and Tables

**Table 1:** Features of the chloroplast genomes of *C. spruceanum* and six Ixoroideae species.

Genome Features	*Calycophyllum spruceanum*	*Coffea arabica*	*Coffea canephora*	*Emmenopterys henryi*	*Fosbergia shweliensis*	*Gardenia jasminoides*	*Scyphiphora hydrophyllacea*
Genome size (bp)	154,480	155,189	154,751	155,379	154,717	154,921	155,132
SSC length (bp)	18,101	18,137	18,133	18,245	18,230	18,095	18,165
LSC length (bp)	84,813	85,166	84,850	85,554	84,747	85,236	85,239
IRA length (bp)	25,783	25,908	23,834	25,790	25,870	25,795	25,864
IRB length (bp)	25,783	25,943	23,884	25,790	25,870	25,795	25,864
No. of protein-coding genes	87	85	86	87	85	87	88
No. of different rRNA genes	4	4	4	4	4	4	4
No. of tRNA genes	37	38	37	37	36	37	37
%GC content in LSC	35.48	31.28	31.75	31.90	35.5	35.3	31.65
%GC content in SSC	31.89	35.35	35.48	35.48	31.4	31.5	35.49
%GC content in IR	43.14	43.01	43.55	43.26	43.2	43.2	43.17

## Data Availability

All data generated during this study are included in this published article.

## Supplementary Materials

The following are available online at https://www.mdpi.com/article/10.3390/genes13010113/s1. Table S1: Genes found in the assembled capirona chloroplast genome. Table S2: The codon-anticodon recognition pattern and codon usage in the chloroplast genome of *Calycophyllum spruceanum*. Table S3: Number of different SSRs types in *C. spruceanum* and six other Ixoroideae species. Figure S1: Reads mapping of the chloroplast genome of *C. spruceanum* reassembled. Figure S2: Matrix of identities of the four regions (LSC, SSC, IRa, IRb) between each of seven chloroplast genomes. Figure S3: Comparison of the genome structure and location of the simple sequence repeat (SSR) of seven Ixoroideae cp genomes, with *C. spruceanum* as a reference. Figure S4: Bayesian phylogenetic tree of 19 species of the Rubiaceae family and outgroup using complete chloroplast genome sequence. Numbers above the branches represent posterior probabilities. Names given to clades refer to subfamilies. The outgroup taxon is *Lonicera hispida*.
