# Supplementary material for: Unlocking the Complete Chloroplast Genome of a Native Tree Species from the Amazon Basin, Capirona (Calycophyllum Spruceanum, Rubiaceae), and Its Comparative Analysis with Other Ixoroideae Species
Source: Genes (Basel). 2022 Jan 7;13(1):113. doi: 10.3390/genes13010113 (PMC8774758; doi:10.3390/genes13010113)
Supplement: Supplementary file 1 [file genes-13-00113-s001.zip › Supplementary figures.pdf]

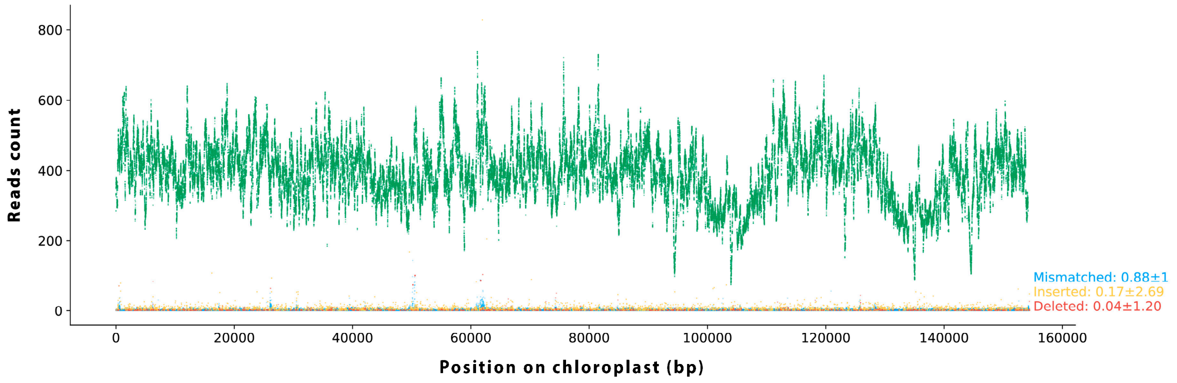

Figure S1. Reads mapping of the chloroplast genome of *C. spruceanum* reassembled.

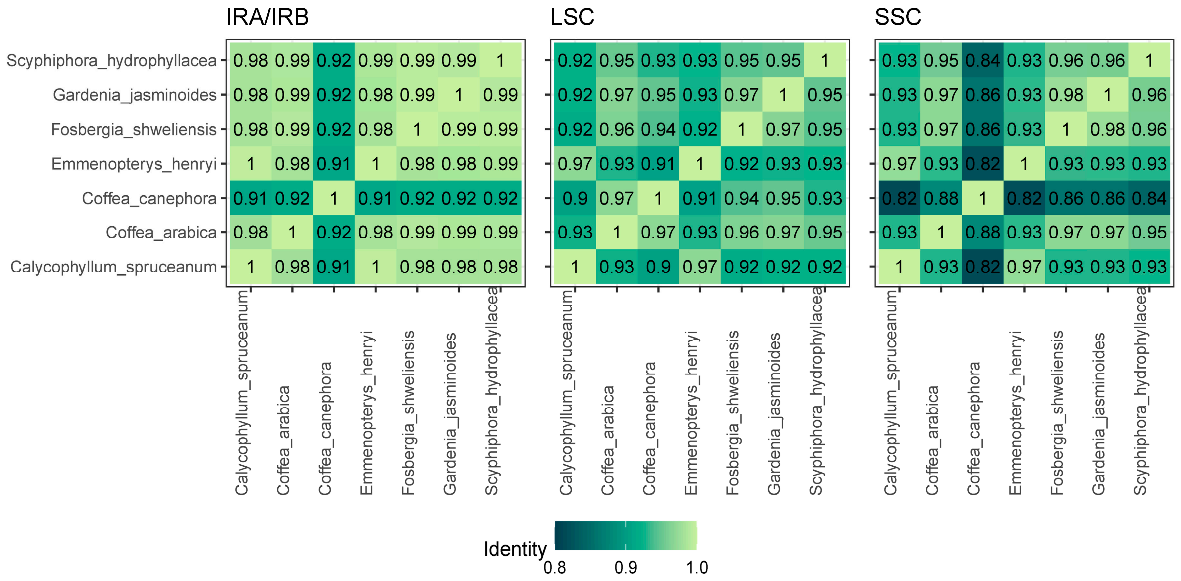

Figure S2. Matrix of identities of the four regions (LSC, SSC, IRa, IRb) between each of seven chloroplast genomes.

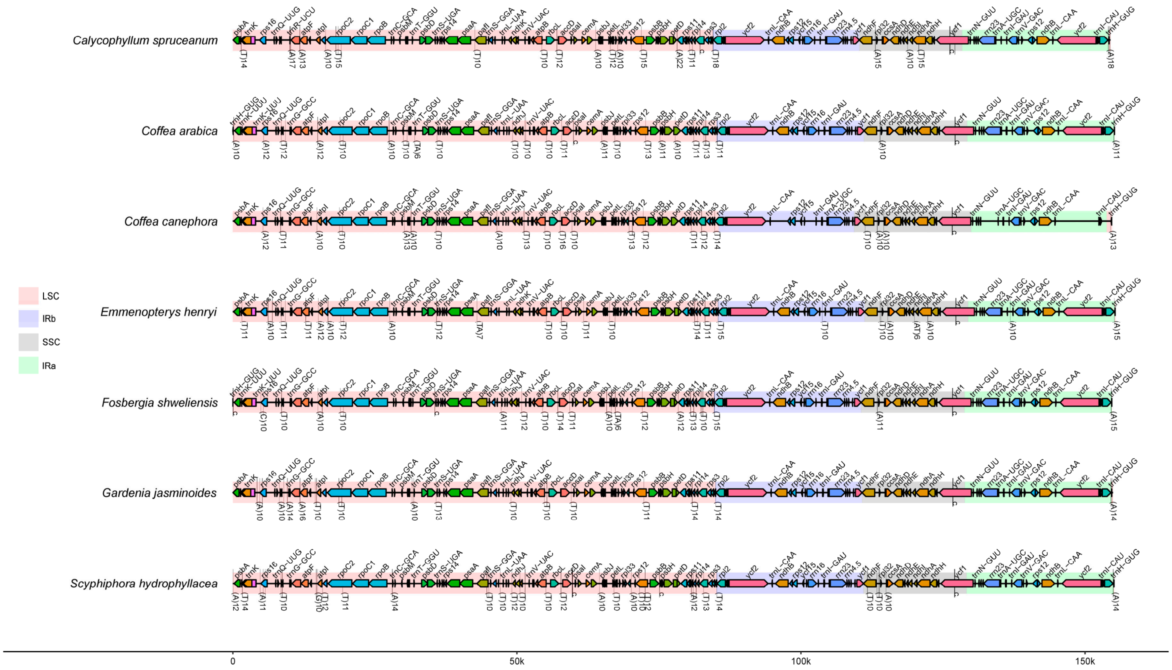

Figure S3. Comparison of the genome structure and location of the simple sequence repeat (SSR) of seven Ixoroideae cp genomes, with *C. spruceanum* as a reference.

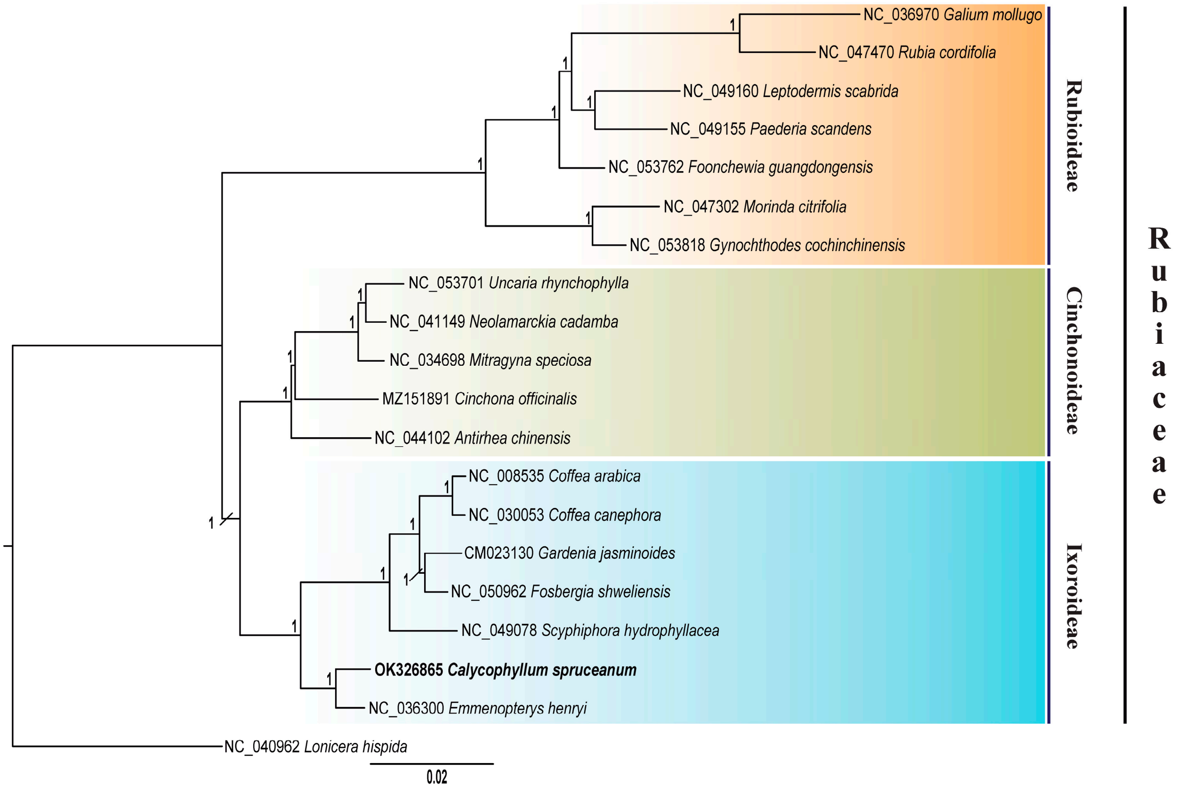

Figure S4. Bayesian phylogenetic tree of 19 species of the Rubiaceae family and outgroup using complete chloroplast genome sequence. Numbers above the branches represent posterior probabilities. Names given to clades refer to subfamilies. The outgroup taxon is *Lonicera hispida*.
